# The Impact of Nutrient Supply on Prostate Cancer Risk Worldwide

**DOI:** 10.3390/nu15245131

**Published:** 2023-12-17

**Authors:** Jinjiang Jiang, Jie Yang, Bo Chen, Jinze Li, Ting Zhang, Daqing Tan, Bo Tang, Qiang Wei

**Affiliations:** 1 Department of Urology, West China Hospital of Sichuan University, No. 37, Guoxue Lane, Chengdu 610041, China; 2021224020118@stu.scu.edu.cn (J.J.); yangjiedoctor666@163.com (J.Y.); yanyuyao1995@gmail.com (B.C.); dr_lijinze@163.com (J.L.); tandaqing1982@163.com (D.T.); btanguro@hotmail.com (B.T.); 2 School of Basic Medicine, Harbin Medical Hospital, Harbin 150000, China; 2021020110@hrbmu.edu.cn

**Keywords:** national supply of nutrients, prostate cancer incidence, animal protein, fat, energy supply

## Abstract

We aim to explore the association between nutrient supply and the incidence of prostate cancer globally. We utilized national nutrient supply data from the Food and Agriculture Organization of the United Nations for 150 countries, including the average supply of total protein (APS), animal protein (AAPS), fat (AFS), animal protein/total protein ratio (ATR), and share of dietary energy supply derived from cereals, roots, and tubers (CR). Prostate cancer incidence data were sourced from the Global Burden Disease 2019 (GBD2019). Correlation, regression analyses, and subgroup analysis were conducted. Our findings imply that incidence of prostate cancer is significantly correlated to APS (ρ = 0.394, *p* < 0.01), AAPS (ρ = 0.560, *p* < 0.01), AFS (ρ = 0.522, *p* < 0.01), ATR (ρ = 0.592, *p* < 0.01), and CR (ρ = −0.667, *p* < 0.01). After adjusting for confounders, regression analysis showed linear relationships between the AAPS (β = 0.605, *p* = 0.006), ATR (β = 70.76, *p* = 0.005), CR (β = −1.4451, *p* < 0.01), and age-standardized incidence rates (ASIRs) of prostate cancer, while no association was observed with APS (β = 0.030, *p* = 0.483) or AFS (β = 0.237, *p* = 0.405). Subgroup analysis suggested that dietary supply indicators were associated with ASIR in middle, middle-high, and high SDI, but not in countries with low and middle-low SDI. In summary, prostate cancer rates globally correlate significantly with AAPS, ATR, and CR, but not with APS and AFS. When considering the SDI of countries, the relationship is generally more pronounced in economically advanced nations, but not evident in low and middle-low SDI countries.

## 1. Introduction

Prostate cancer is a common global health concern, with approximately 1.4 million new cases and 375,000 deaths in 2020 [1]. Its precise causes remain unclear, but genetic and environmental factors, including nutrition, are believed to play roles. Notably, the higher incidence of prostate cancer among Asian immigrants in Western countries compared to their native counterparts suggests that environmental changes, including dietary shifts and improved healthcare, might contribute to this rise [2,3]. Emerging evidence underscores the influence of nutrition on prostate cancer development and progression. For instance, a meta-analysis by Alzahrani [4] found a dose–response relationship between dairy protein consumption and prostate cancer risk. Another study spanning 24 years linked dietary α-linolenic acid intake to lethal prostate cancer [5]. Abnormal lipid metabolism can drive hormone-sensitive prostate cancer to castration-resistant prostate cancer [6]. In a mouse model, increased fat intake lowers histone methylation through enhanced MYC transcription, promoting prostate cancer cell proliferation [7]. However, a pooled study incorporating 14 cohort studies did not establish linear associations between prostate cancer risk and fat intake, including saturated, polyunsaturated, or monounsaturated fats [8]. Discrepancies in epidemiological findings can be attributed in part to variations in research settings among different countries, including differences in data quality and collection criteria. Recognizing that national nutrient supply closely approximates actual dietary intake [9], our study examines the relationship between prostate cancer incidence and national-level nutrient supply to shed light on global dietary factors and their impact on prostate cancer risk.

## 2. Materials and Methods

### 2.1. Data Resources

The prostate cancer incidence data for this study were sourced from the GBD 2019 study online database, accessible at http://ghdx.healthdata.org/gbd-results-tool (accessed on 23 March 2023). The Global Burden of Disease 2019 (GBD 2019) project is managed by the Institute for Health Metrics and Evaluation (IHME) and offers comprehensive national estimates, encompassing incidence, mortality, and prevalence, stratified by cancer type and gender across 204 countries/territories [10]. The IHME also makes available socio-demographic index (SDI) and Healthcare Access and Quality Index (HAQ) data, which can be accessed on the same website. Data for the dietary variables were obtained from United Nations Food and Agriculture Organization database (http://www.fao.org/faostat/), which provides a comparative assessment of food accessibility across 195 countries. We collected five dietary variables, including average protein supply (g/cap/day) (APS), average supply of protein from animal sources (g/cap/day) (AAPS), average fat supply (g/cap/day) (AFS), and the proportion of dietary energy supply originating from cereals, roots, and tubers (%) (CR) from United Nations Food and Agriculture Organization database from 2014 to 2016.

### 2.2. Risk Factors

We assessed national development using the socio-demographic index (SDI), which considers per capita income, years of education beyond age 15, and general fertility rates [11]. The SDI scores range from 0 to 1, with higher scores indicating greater development. Furthermore, in order to address the differences in medical care and screening across countries, we factored in the Healthcare Access and Quality Index (HAQ) from the IHME [12]. HAQ evaluates healthcare quality and accessibility across diverse countries, drawing from data sources such as surveys, vital registration systems, and medical records with scores ranging from 0 to 100, higher scores signifying better healthcare quality and access. Lastly, median age data for each country were obtained from the United Nations’ Department of Economic and Social Affairs dataset [13]. Age strongly correlates with total prostate cancer risk [14], particularly after age 55, as noted by Ferlay et al. [15].

### 2.3. Statistical Analysis

As a previous study [16] described, age-standardized incidence rates (ASIRs) were obtained to estimate the overall trend of prostate cancer incidence. Animal protein/total protein ratio (ATR) was calculated as the AAPS divided by APS. 

We conducted correlation tests and regression analysis to obtain effective indicators, such as Spearman correlation coefficients (ρ), simple linear regression coefficients (β), and two-sided p-values. To eliminate confounding effects, regression analyses, adjusted for covariates like median age, SDI, and HAQ, were employed.

We performed data visualization and statistical analyses using R Studio (Version 4.1.3, R core team) and SPSS Base (Version 26.0) for Windows.

## 3. Results

### 3.1. Characteristic Description of Nutritional Supply and Prostate Cancer Incidence by Countries

A dataset encompassing 150 countries is presented in Table 1, detailing their key attributes such as geographic location, national nutrient supply, SDI, and ASIR. Compared to other regions, APS, AAPS, and ATR in Europe were observed to be higher and peaked in Western Europe. Specifically, Iceland possessed the highest APS (138.7 g/cap/day), AAPS (99.3 g/cap/day), and ATR (0.72). For countries in the Americas and Oceania, there was a high supply of animal protein in several countries, including the United States, Canada, Australia, and New Zealand, where their ATR was greater than 0.50. Conversely, the APS, AAPS, and ATR were at a relatively low level in most countries in East Asia, South Asia, and Africa. Most countries in Africa had an APS of no more than 80 g/cap/day, and a majority of countries had an ATR below 0.40. While in most countries, APS, AAPS, and ATR changed in parallel, some, like Mongolia, had a higher ATR (0.66) despite relatively lower levels of AAPS (58.7 g/cap/day) and APS (88.7 g/cap/day). 

Moreover, a notable observation is the peak in AFS in Western Europe, exemplified by Belgium’s highest AFS at 164.0 g/cap/day, with the majority of countries in this region exceeding an average fat supply of 120 g/cap/day. In North America and Australasia, specifically the United States, Canada, Australia, and New Zealand, AFS levels significantly exceeded those of other countries within the same continent. Conversely, in Africa and Asia, most nations fell within the range of 50 to 80 g/cap/day, with only a limited number surpassing an average fat supply of 100 g/cap/day. 

Evident differences in CR were observed between East and West. The overall level of CR in Europe, Australasia, and North America is lower in comparison with a higher level in Africa and Asia, where most African countries have a CR of over 60%. In particular, Madagascar in Africa had the highest CR (79%). 

According to the results of GBD, prostate cancer incidence is generally higher in Europe and America and lower in Asia. In Europe, Western and Eastern Europe had higher incidence rates than Central Europe, and the country with the highest incidence rate was Estonia (ASIR 122.97), together with a high level of SDI (0.82) and HAQ (81.4). Most American countries have higher prostate cancer rates, with the Caribbean region as a peak. Dominica, in Andean Latin America, had the world’s highest prostate cancer incidence and an SDI of 0.72. Prostate cancer incidence in Asian countries is generally low, with an ASIR below 30. Of note is that the regional disparity of ASIR can be found in Asia, where South and East Asia are lower than in Central Asia and the high-income Asia–Pacific. Moreover, the incidence of prostate cancer in African countries included in our study varied from 20 to 60.

### 3.2. Correlation Analysis between Incidence of Prostate Cancer and Nutritional Supply Worldwide

**Figure 1:**
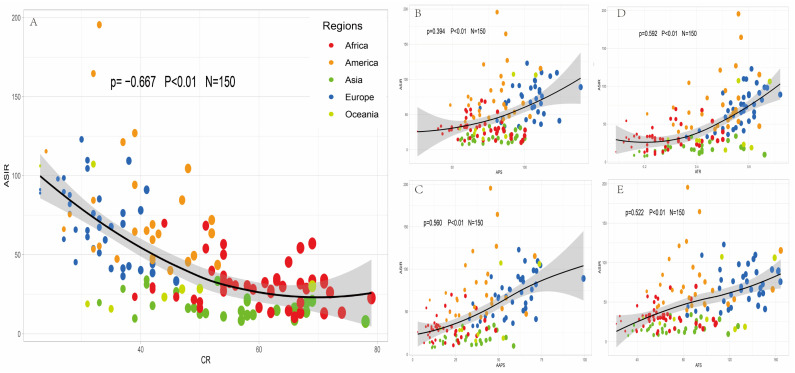
Correlation analysis between the incidence of prostate cancer and nutrient supply in 150 countries. (**A**) CR is inversely correlated with ASIR, ρ = −0.667, *p* < 0.01. (**B**) APS is positively correlated with ASIR, ρ = 0.394, *p* < 0.01. (**C**) AAPS is positively correlated with ASIR, ρ = 0.560, *p* < 0.01. (**D**) ATR is positively correlated with ASIR, ρ = 0.592, *p* < 0.01. (**E**) AFS is positively correlated with ASIR, ρ = 0.522, *p* < 0.01. APS, average protein supply; AAPS, average supply of protein of animal origin; ATR, animal protein/total protein ratio; APS, average fat supply; CR, share of dietary energy supply derived from cereals, roots and tubers; ASIR, age standardized incidence rate.

Correlation analysis between the incidence of prostate cancer and nutrition supply at a national level was employed, and is presented in Figure 1. An inverse correlation (ρ = −0.667, *p* < 0.01) exists between CR and ASIR. European and American countries cluster on the left side with lower CR and higher ASIR, while Asia and Africa are on the right side with higher CR and lower ASIR. According to the results of Figure 1B–E, we can observe a positive correlation between prostate cancer incidence and APS (ρ = 0.394, *p* < 0.01), AAPS (ρ = 0.560, *p* < 0.01), ATR (ρ = 0.592, *p* < 0.01), and AFS (ρ = 0.522, *p* < 0.01) globally. In essence, countries with a higher AFS, APS, AAPS, and ATR tend to exhibit a relatively higher incidence of prostate cancer. European countries, known for their high fat and protein supply, are mostly on the right side of the curve, while other regions’ countries are more evenly distributed along the curve.

### 3.3. Regression Analysis of Nutritional Supply and Prostate Cancer Incidence

Regression analyses were performed to explore global associations between nutritional supply and prostate cancer incidence, as shown in Table 2. In the crude model, it is evident that all factors, including APS (β = 0.669, *p* < 0.01), AAPS (β = 0.939, *p* < 0.01), ATR (β = 120.86, *p* < 0.01), AFS (β = 0.447, *p* < 0.01), and CR (β = −1.49, *p* < 0.01), exhibit linear relationships with ASIR. However, upon adjusting for confounding variables like median age, SDI, and HAQ, AAPS (β = 0.605, *p* = 0.006), ATR (β = 70.76, *p* = 0.005), and CR (β = −1.4451, *p* < 0.01) remain linearly associated with ASIR. Conversely, APS (β = 0.030, *p* = 0.483) and AFS (β = 0.237, *p* = 0.405) do not exhibit statistical significance. 

### 3.4. Subgroup Analysis of Nutritional Supply and Prostate Cancer Incidence Based on SDI Level

We performed an extensive subgroup analysis stratified by SDI, and the results are presented comprehensively in Table 3. Notably, in high-SDI countries, increased APS (β = 0.891, *p* < 0.043) and AAPS (β = 1.079, *p* = 0.011) are significantly associated with higher ASIR. Additionally, ASIR tends to increase with higher ATR in both high- (β = 271.24, *p* = 0.003) and middle-SDI (β = 125.29, *p* = 0.019) countries. Interestingly, we found no significant correlation between AFS and ASIR across all SDI classifications. The subgroup analysis for CR revealed that ASIR increased with CR in middle- (β = −1.699, *p* = 0.013), high-middle- (β = −3.267, *p* = 0.009), and high-SDI (β = −2.493, *p* = 0.023) countries. 

## 4. Discussion

APS demonstrated no significant association with prostate cancer ASIR across all countries, yet a correlation emerged in high-SDI countries (β = 0.891, *p* = 0.043). This finding resonates with Alzahrani’s study [4], which indicated that the consumption of dairy-derived protein rather than total protein intake elevate prostate cancer risk. The strong correlation in high SDI countries is due to their higher overall protein supply compared to lower-SDI nations. Additionally, the influence of protein sources, encompassing both animal- and plant-derived proteins, can fluctuate and exert divergent effects on prostate cancer development. Therefore, considering preferences for animal or plant protein in these countries is more appropriate.

Our findings indicate that a 1 g increase in AAPS per individual per day corresponds to a 0.6 per 100,000 population rise in ASIR of prostate cancer on a global scale. Animal protein intake is reportedly associated with promoting prostate cancer due to excessive insulin-like growth factor-1 (IGF-1) production in the bloodstream, which plays a central role in prostate cancer growth and invasion through a complex network involving integrin–FAK signaling and the Akt-mTOR pathway [17,18]. A large meta-analysis of 324,197 participants found that animal protein intake does not significantly increase the risk of prostate cancer in the general population, with a relative risk of 0.99 (95% CI: 0.95–1.04) [4]. Interestingly, the significant association between the AAPS and ASIR of prostate cancer manifested exclusively within high-SDI countries, not in countries with different economic levels. It is plausible that countries of other economic levels exhibit AAPS values that are not as high as those observed in high-SDI countries. Particularly, one must also take into account the proportion of animal protein in relation to the total daily protein supply. Especially when animal protein intake forms a small proportion of daily protein intake, its impact may differ. 

Our regression analysis showed that a 0.014 increase in ATR is linked to a 1 per 100,000 population increase in ASIR of prostate cancer, consistently in middle- and high-SDI countries. Crucially, ATR also gives insights into other protein origins, including plant-based sources. The consumption of plant protein from plant-based foods introduces a substantial quantity of fiber, antioxidants, and phytoestrogens with potential anti-cancer effects [19]. Therefore, it is plausible that a plant-based diet might better capture the impact of ATR on the incidence of prostate cancer. This perspective is in alignment with the outcomes of a prior prospective cohort study, which reported that adopting a healthy plant-based diet was linked to reduced risks of total (HR = 0.84, *p* = 0.046) and lethal prostate cancer (HR = 0.56, *p* = 0.03) among individuals aged <65 [20].

Given the relationship between ASIR and AAPS, and ATR, the incongruity between our results and genuine consumption patterns implies that the national protein supply might have been misconstrued as daily intake in the past, amplifying the recognition of the association between animal protein and elevated prostate cancer risk.

A substantial body of research has highlighted the role of aberrant fat metabolism in driving the initiation and progression of prostate cancer. For instance, Liss et al. conducted a decade-long study involving 1903 men, and established an elevated risk of prostate cancer associated with dietary fat intake [21]. In murine models, a reduced fat diet led to lower serum prostate-specific antigen levels and slower tumor growth in LAPC4 xenografts compared to a high-fat diet [22]. High-fat diets can induce localized inflammation in the prostate gland, promoting prostate cancer growth by suppressing the tumor immune response, potentially through the IL-6 and STAT3 signaling pathway [23]. Furthermore, inflammation induced by tumors and related cytokines is recognized as a factor in prostate cancer development and progression. In vitro studies on both androgen-sensitive and androgen-insensitive prostate cancer cell lines indicate that reducing prostate inflammation with antioxidant-rich herbal extracts leads to tumor apoptosis via a specific mitochondrial pathway [24]. In our study, regression analysis showed that the AFS is not significantly associated with the ASIR of prostate cancer (*p* = 0.237, *p* = 0.405) after adjusting for median age, SDI, and HAQ. These results are consistent with a meta-analysis of fourteen cohort studies, which also found no clear connection between total fat intake and prostate cancer risk. One plausible explanation for this might stem from the intricate composition of dietary fat sources, encompassing saturated and unsaturated fat, trans-fatty acids, and cholesterol. Analogous to the complexity seen in daily protein consumption, diverse fat types exhibit distinct pathogenic influences. Saturated fatty acids may reduce prostate cancer patient survival [25], while unsaturated fatty acids found in fish and vegetable oils are associated with a reduced risk of prostate cancer [26]. Taken together, our investigation indicates an unclear association between prostate cancer incidence and either the average total fat supply or actual fat consumption. The regional economic disparities, dietary preferences, and the specific types of fat consumed might contribute to these results, highlighting the importance of considering such nuances in future research. 

Energy restriction can potentially inhibit prostate tumor growth and reduce the expression of growth factors like vascular endothelial growth factor and insulin-like growth factor in tumor models [27], suggesting a role for dietary energy sources in tumor growth regulation. A noteworthy discovery was made: a 1.45-unit increase in CR corresponded to a reduction of 1 per 100,000 in the ASIR of prostate cancer in our study. Additionally, a significant decrease in the ASIR of prostate cancer was observed with increasing CR in middle-high- and high-SDI countries. Our results suggest that cereals, roots, and tubers are less risky energy sources compared to fat and protein, considering that carbohydrates are the primary daily energy source. In alignment with our findings, Hsieh et al. reported a positive association between total energy intake and prostate cancer risk, with the odds ratio of the highest quintile compared to the lowest quintile reaching 3.79 (*p* = 0.002) [28]. Consequently, considering an equivalent energy supply, substituting carbohydrates as energy sources instead of protein and fat could potentially lead to a reduction in the incidence of prostate cancer. Nonetheless, it is advisable to contemplate carbohydrate reduction when alternative energy sources confer greater beneficial effects than carbohydrates [29]. Moreover, in conjunction with the consumption of cereals, roots, and tubers, the incorporation of plant-based constituents such as fiber, vitamins, minerals, and phytochemicals has been demonstrated to mitigate the risk of prostate cancer, as expounded upon earlier.

Interestingly, no significant correlations were observed between the aforementioned five diet-related factors and prostate cancer incidence in low- and low-middle-SDI countries, possibly due to their limited overall food supply. This prompts the question of whether the dietary traits associated with an increased risk of prostate cancer are linked to greater economic prosperity. This raises the issue of whether the “wealthier” dietary traits associated with increased prostate cancer risk. Additionally, it has been proposed that nations with less advanced economic development may exhibit questionable reliability in their tumor registration center data, potentially leading to underestimations or misclassifications of national disease incidence. 

In a broader context, definitive conclusions about the singular role of a particular nutrient as a causal factor for prostate cancer are challenging to establish. This arises from the inherent difficulty in precisely quantifying the intake of individual nutrients, compounded by the inevitable collinearity among various nutrients. Consequently, it becomes imperative to consider both the interplay of dietary nutrient proportions and the intricate nature of dietary sources when assessing the multifaceted effects of diverse nutrients on cancer development.

Regarding limitations, our regression model lacked consideration of genetic factors, dietary preferences, income disparities, nutrient distribution, and cancer screening levels, possibly introducing bias. Secondly, previous studies have confirmed that the national nutrition supply was pretty close to the actual consumption of specific nutrition through three national databases, and nutritional availability in our study can partially reflect the consumption [9]. Nonetheless, these data have not been testified in a wider range of countries, and the gap between nutrient intake and supply of nutrients may be mis-estimated. Variations in nutrient intake due to social and economic disparities limit our findings to a national-level correlation, challenging their applicability to understanding individual nutrient intake and cancer occurrence. Finally, the precision of the data sourced from the database may introduce minor imperfections in the results.

## 5. Conclusions

Our study shows a positive correlation between national provisions of AAPS and ATR in different countries, and prostate cancer incidence. Yet, no statistically significant associations were found for AFS and APS. On the other hand, a lower risk of prostate cancer was linked to increased CR. Finally, dietary factors’ influences on prostate cancer risk show a consistent pattern across middle- to high-SDI countries, but not in low- and middle-low-SDI ones. Additional research on these clinically significant issues is urgently needed in the near future.

## Figures and Tables

**Table 1:** Age-standardized incidence of prostate cancer in 2015 among 150 countries, with characteristics of nutrient supply.

Regions	Nation	APS (g/cap/day)	AAPS (g/cap/day)	ATR	AFS (g/cap/day)	CR (%)	SDI	ASIR (per 100,000)
Europe								
	Central Europe							
	Albania	111.3	58.3	0.52	112.3	37	0.66	36.35
	Bosnia and Herzegovina	96.0	35.0	0.36	83.6	46	0.70	33.18
	Bulgaria	83.7	39.3	0.47	90.7	42	0.75	38.44
	Croatia	86.7	50.0	0.58	111.3	33	0.78	67.30
	Czechia	87.3	49.7	0.57	130.0	31	0.82	65.33
	Hungary	85.0	46.7	0.55	144.7	29	0.78	45.24
	Montenegro	109.3	65.7	0.60	136.7	32	0.78	53.89
	North Macedonia	81.0	33.0	0.41	101.0	37	0.73	41.36
	Poland	101.0	54.3	0.54	121.3	38	0.79	42.70
	Romania	106.7	53.3	0.50	119.0	40	0.74	40.18
	Serbia	84.7	42.3	0.50	84.0	42	0.75	44.16
	Slovakia	69.3	35.0	0.51	121.3	34	0.80	59.34
	Slovenia	97.7	52.0	0.53	112.7	40	0.83	78.06
	Eastern Europe							
	Belarus	89.0	48.0	0.54	130.0	37	0.73	69.49
	Estonia	101.7	61.7	0.61	115.0	30	0.82	122.97
	Latvia	98.3	56.3	0.57	123.0	37	0.81	76.34
	Lithuania	122.3	72.7	0.59	96.0	38	0.82	109.41
	Ukraine	86.3	37.7	0.44	83.0	46	0.73	33.38
	Western Europe							
	Austria	107.3	64.0	0.60	162.3	27	0.84	89.79
	Belgium	98.3	56.7	0.58	164.0	28	0.84	75.78
	Cyprus	91.7	45.7	0.50	111.7	41	0.83	91.07
	Denmark	110.3	71.7	0.65	131.7	28	0.88	81.98
	Finland	117.7	74.3	0.63	140.7	31	0.84	104.58
	France	108.0	63.3	0.59	148.7	33	0.82	72.61
	Germany	104.3	62.7	0.60	143.3	28	0.89	87.78
	Greece	106.7	58.0	0.54	149.0	31	0.79	61.20
	Iceland	138.7	99.3	0.72	162.0	23	0.85	89.00
	Ireland	110.3	64.3	0.58	134.0	33	0.85	85.26
	Israel	124.0	71.7	0.58	150.7	35	0.79	41.20
	Italy	104.0	54.3	0.52	146.7	35	0.79	67.71
	Luxembourg	111.3	69.0	0.62	138.7	29	0.89	65.81
	Malta	113.3	64.3	0.57	115.0	33	0.78	51.13
	Netherlands	107.3	72.0	0.67	128.0	26	0.87	97.94
	Norway	111.7	65.3	0.58	142.3	31	0.90	109.78
	Portugal	112.0	69.3	0.62	135.0	32	0.73	76.09
	Spain	105.3	65.7	0.62	145.7	27	0.76	59.77
	Sweden	107.0	70.3	0.66	136.0	27	0.86	98.56
	Switzerland	96.3	61.3	0.64	156.7	23	0.92	91.19
Asia								
	Central Asia							
	Armenia	97.0	44.3	0.46	95.3	41	0.68	31.18
	Azerbaijan	90.3	31.3	0.35	64.0	58	0.67	20.67
	Georgia	75.7	29.0	0.38	68.7	53	0.83	33.38
	Kazakhstan	94.7	54.3	0.57	127.3	33	0.71	19.62
	Kyrgyzstan	84.0	34.3	0.41	65.3	54	0.58	10.94
	Mongolia	88.7	58.7	0.66	118.8	39	0.59	9.68
	Tajikistan	65.7	17.7	0.27	62.0	59	0.52	11.98
	Turkmenistan	91.3	37.0	0.41	81.0	58	0.65	11.70
	Uzbekistan	98.7	40.0	0.41	89.7	50	0.61	12.54
	High-income Asia Pacific							
	Japan	86.0	47.3	0.55	86.7	41	0.86	34.11
	Republic of Korea	93.7	49.0	0.52	111.3	37	0.54	28.34
	South Asia							
	Bangladesh	57.0	11.3	0.20	32.3	78	0.45	7.78
	India	61.7	13.0	0.21	54.3	57	0.53	8.40
	Nepal	71.3	11.7	0.16	58.3	66	0.39	8.78
	Pakistan	64.3	27.0	0.42	76.0	51	0.42	12.95
	East Asia							
	China	100.1	39.3	0.39	98.2	50	0.66	16.09
	Democratic People’s Republic of Korea	55.7	10.0	0.18	36.0	68	0.86	13.83
	Southeast Asia							
	Cambodia	65.0	18.7	0.29	37.3	69	0.44	20.75
	Indonesia	63.7	19.3	0.30	51.4	68	0.63	19.39
	Lao People’s Democratic Republic	75.3	17.7	0.24	43.7	63	0.46	14.78
	Malaysia	78.0	45.0	0.58	91.0	42	0.72	17.74
	Mauritius	86.0	39.0	0.45	91.3	46	0.69	26.53
	Myanmar	93.7	42.7	0.46	82.0	49	0.49	16.30
	Philippines	60.7	25.3	0.42	53.4	58	0.60	22.07
	Sri Lanka	65.0	17.7	0.27	48.7	57	0.67	14.37
	Thailand	61.3	25.7	0.42	61.7	48	0.67	16.18
	Timor-Leste	56.7	16.0	0.28	50.0	66	0.50	16.12
	Vietnam	81.7	30.7	0.38	72.7	60	0.59	16.52
Africa								
	North Africa and Middle East							
	Afghanistan	58.6	12.0	0.20	39.7	71	0.31	12.89
	Algeria	90.7	26.3	0.29	97.0	50	0.63	16.24
	Egypt	97.6	23.7	0.24	57.3	66	0.63	10.41
	Iran	85.7	25.0	0.29	77.7	54	0.65	26.67
	Iraq	62.3	13.3	0.21	72.4	60	0.63	16.58
	Jordan	72.3	24.7	0.34	91.0	46	0.71	23.19
	Kuwait	100.3	48.3	0.48	106.0	42	0.83	28.02
	Lebanon	70.3	22.6	0.32	90.9	44	0.69	69.87
	Morocco	98.7	26.7	0.27	68.0	62	0.52	13.43
	Oman	85.3	43.0	0.50	76.6	42	0.76	29.73
	Saudi Arabia	91.0	37.3	0.41	107.6	49	0.78	21.61
	Tunisia	100.0	28.3	0.28	94.3	50	0.65	19.95
	United Arab Emirates	80.7	32.3	0.40	108.3	39	0.87	23.30
	Yemen	53.9	11.3	0.21	39.3	65	0.41	14.59
	Eastern Sub-Saharan Africa							
	Comoros	54.7	14.3	0.26	62.0	56	0.43	30.46
	Djibouti	69.0	15.0	0.22	63.3	54	0.43	33.83
	Ethiopia	71.4	6.7	0.09	29.7	74	0.30	13.34
	Kenya	62.7	15.0	0.24	41.7	59	0.48	28.04
	Madagascar	43.3	9.0	0.21	22.0	79	0.37	22.61
	Malawi	67.4	10.0	0.15	45.4	67	0.36	19.60
	Rwanda	59.3	7.7	0.13	25.0	54	0.40	32.27
	United Republic of Tanzania	60.0	11.0	0.18	54.0	55	0.39	32.16
	Zambia	59.3	13.7	0.23	50.0	69	0.47	33.71
	Southern Sub-Saharan Africa							
	Botswana	72.7	30.7	0.42	68.7	51	0.61	68.31
	Eswatini	59.3	19.0	0.32	56.0	54	0.56	56.62
	Lesotho	62.7	19.0	0.30	49.0	69	0.48	57.09
	Namibia	63.0	21.7	0.34	54.7	54	0.59	49.99
	South Africa	81.3	37.0	0.46	86.7	51	0.66	53.89
	Western Sub-Saharan Africa							
	Benin	67.0	16.0	0.24	51.0	67	0.32	31.63
	Burkina Faso	79.7	11.0	0.14	63.3	64	0.24	29.77
	Cameroon	72.7	12.7	0.17	56.7	54	0.46	36.65
	Chad	76.7	25.3	0.33	65.0	62	0.22	27.40
	Cote d’Ivoire	58.4	13.3	0.23	47.7	71	0.38	32.30
	Ghana	59.6	14.0	0.23	40.0	65	0.52	45.33
	Guinea-Bissau	42.3	9.0	0.21	62.3	63	0.33	33.54
	Liberia	40.3	11.0	0.27	58.0	64	0.34	29.85
	Mali	80.0	19.0	0.24	58.0	67	0.24	13.15
	Mauritania	82.0	30.0	0.37	68.6	54	0.47	30.61
	Niger	81.1	10.0	0.12	50.7	62	0.14	25.97
	Nigeria	60.0	8.0	0.13	58.0	67	0.49	54.30
	Senegal	60.4	14.0	0.23	66.3	63	0.36	34.71
	Sierra Leone	49.6	12.0	0.24	50.0	68	0.32	28.51
	Togo	56.0	9.0	0.16	51.0	71	0.39	34.11
	Central Sub-Saharan Africa							
	Angola	54.3	17.9	0.33	59.9	58	0.43	30.42
	Central African Republic	48.0	20.0	0.42	54.7	54	0.26	27.58
	Congo	49.7	22.7	0.46	44.8	61	0.34	32.29
	Democratic Republic of the Congo	26.0	3.0	0.12	26.0	72	0.54	26.08
	Gabon	84.3	42.0	0.50	54.6	52	0.62	39.78
America								
	High-income North America							
	Canada	99.0	54.3	0.55	157.3	27	0.86	66.09
	United States of America	111.0	71.0	0.64	164.3	24	0.85	115.42
	Southern Latin America							
	Argentina	103.7	66.0	0.64	118.7	36	0.69	47.25
	Chile	87.0	46.0	0.53	90.3	42	0.74	59.48
	Uruguay	85.7	46.0	0.54	105.3	39	0.68	64.56
	Andean Latin America							
	Ecuador	64.6	33.3	0.52	98.6	42	0.62	46.65
	Peru	85.3	39.7	0.47	57.3	53	0.63	43.49
	Caribbean							
	Barbados	87.0	49.3	0.57	94.0	32	0.73	164.64
	Belize	68.7	25.7	0.37	70.4	41	0.59	65.17
	Cuba	83.7	30.7	0.37	67.7	48	0.65	104.56
	Dominica	81.0	45.3	0.56	83.7	33	0.72	195.56
	Dominican Republic	61.0	30.3	0.50	98.7	28	0.57	75.49
	Guyana	84.0	37.0	0.44	62.0	47	0.60	84.51
	Haiti	48.7	10.0	0.21	48.3	52	0.42	63.52
	Jamaica	71.7	35.0	0.49	76.3	37	0.67	121.25
	Saint Vincent and the Grenadines	88.0	48.7	0.55	82.7	39	0.61	126.95
	Suriname	61.3	26.0	0.42	78.3	43	0.62	63.14
	Trinidad and Tobago	85.3	43.7	0.51	88.3	39	0.75	94.19
	Central Latin America							
	Colombia	68.7	36.0	0.52	85.0	33	0.61	55.39
	Costa Rica	76.7	42.7	0.56	94.0	32	0.66	84.40
	El Salvador	75.3	27.0	0.36	58.7	48	0.55	45.57
	Guatemala	69.0	21.7	0.31	62.7	49	0.50	49.33
	Honduras	59.0	19.7	0.33	83.0	45	0.48	39.95
	Mexico	89.3	43.0	0.48	98.3	42	0.63	47.82
	Nicaragua	63.0	19.3	0.31	60.0	52	0.50	71.91
	Panama	78.7	43.0	0.55	80.0	42	0.66	69.11
	Tropical Latin America							
	Brazil	93.0	52.0	0.56	124.0	32	0.62	53.49
	Paraguay	65.0	25.0	0.38	87.3	48	0.62	45.46
Oceania								
	Oceania							
	Fiji	71.7	28.7	0.40	72.0	50	0.64	28.44
	Kiribati	71.3	36.7	0.51	124.7	35	0.51	15.73
	Papua New Guinea	65.3	38.0	0.58	59.6	44	0.38	23.12
	Samoa	87.0	52.7	0.61	133.3	31	0.63	18.82
	Solomon Islands	55.3	16.3	0.29	45.3	69	0.39	29.99
	Vanuatu	66.0	27.3	0.41	100.3	47	0.47	28.18
	Australasia							
	Australia	107.7	73.7	0.68	159.0	23	0.83	106.18
	New Zealand	92.3	51.3	0.56	113.3	32	0.83	107.29

**Table 2:** Regression analysis of nutritional supply and prostate cancer incidence at a national level.

Incidence of Prostate Cancer				
	Crude Model		Adjusted Model	
	β (±SD)	*p* Value	β (±SD)	*p* Value
APS	0.669 (±0.12)	<0.01	−0.030 (±0.194)	0.483
AAPS	0.939 (±0.11)	<0.01	0.605 (±0.215)	0.006
ATR	120.86 (±14.34)	<0.01	70.76 (±24.68)	0.005
AFS	0.447 (±0.07)	<0.01	0.237 (±0.11)	0.405
CR	−1.49 (±0.15)	<0.01	−1.451 (±0.30)	<0.01

APS, average protein supply; AAPS, average supply of protein of animal origin; ATR, animal protein/total protein ratio; APS, average fat supply; CR, share of dietary energy supply derived from cereals, roots and tubers; β, regression coefficient; SD, standard deviation.

**Table 3:** Subgroup analysis of nutritional supply and prostate cancer incidence based on SDI.

	Crude Model		Adjusted Model	
	β (±SD)	*p* Value	β (±SD)	*p* Value
APS				
Low SDI	−0.0324 (±0.18)	0.076	−0.246 (±0.22)	0.275
Low-middle SDI	−0.328 (±0.273)	0.239	−0.416 (±0.369)	0.27
Middle SDI	−0.380 (±0.422)	0.375	−0.662 (±0.417)	0.125
High-middle SDI	0.232 (±0.532)	0.666	0.183 (±0.576)	0.754
High SDI	1.184 (±0.338)	0.002	0.891 (±0.414)	0.043
AAPS				
Low SDI	−0.180 (±0.309)	0.564	−0.584 (±0.293)	0.058
Low-middle SDI	−0.257 (±0.334)	0.447	−0.697 (±0.50)	0.176
Middle SDI	−0.904 (±0.524)	0.095	0.583 (±0.558)	0.306
High-middle SDI	0.307 (±0.568)	0.593	0.596 (±0.578)	0.313
High SDI	1.225 (±0.228)	<0.01	1.079 (±0.388)	0.011
ATR				
Low SDI	2.498 (±19.303)	0.898	−31.475 (±20.199)	0.133
Low-middle SDI	−4.944 (±33.371)	0.883	−46.748 (±50.242)	0.361
Middle SDI	153.726 (±48.558)	0.004	125.29 (±49.966)	0.019
High-middle SDI	125.965 (±84.489)	0.147	123.216 (±88.264)	0.175
High SDI	241.99 (±50.08)	<0.01	271.24 (±81.122)	0.003
AFS				
Low SDI	0.150 (±0.160)	0.355	0.057 (±0.156)	0.717
Low-middle SDI	−0.112 (±0.172)	0.518	−0.45 (±0.259)	0.095
Middle SDI	0.069 (±0.286)	0.811	−0.247 (±0.335)	0.468
High-middle SDI	−0.134 (±0.305)	0.665	0.157 (±0.371)	0.676
High SDI	0.479 (±0.191)	0.019	0.244 (±0.300)	0.425
CR				
Low SDI	−0.569 (±0.234)	0.022	−0.300 (±0.268)	0.274
Low-middle SDI	−0.434 (±0.365)	0.244	−0.417 (±0.6)	0.493
Middle SDI	−1.722(±0.521)	0.003	−1.699 (±0.638)	0.013
High-middle SDI	−2.796 (±1.124)	0.019	−3.267 (±1.156)	0.009
High SDI	−2.246 (±0.594)	0.001	−2.493 (±1.020)	0.023

APS, average protein supply; AAPS, average supply of protein of animal origin; ATR, animal protein/total protein ratio; APS, average fat supply; CR, share of dietary energy supply derived from cereals, roots and tubers; β, regression coefficient; SD, standard deviation.

## Data Availability

Data and materials can be provided upon request with the corresponding author.
